# Applications of water molecules for analysis of macromolecule properties

**DOI:** 10.1016/j.csbj.2020.02.001

**Published:** 2020-02-12

**Authors:** Karolina Mitusińska, Agata Raczyńska, Maria Bzówka, Weronika Bagrowska, Artur Góra

**Affiliations:** Tunneling Group, Biotechnology Centre, Silesian University of Technology, Krzywoustego 8, Gliwice, Poland

**Keywords:** Water molecules, Water placement, Macromolecule structure, Transportation, Water sites, Tunnel detection

## Abstract

Water molecules maintain proteins’ structures, functions, stabilities and dynamics. They can occupy certain positions or pass quickly *via* a protein’s interior. Regardless of their behaviour, water molecules can be used for the analysis of proteins’ structural features and biochemical properties. Here, we present a list of several software programs that use the information provided by water molecules to: i) analyse protein structures and provide the optimal positions of water molecules for protein hydration, ii) identify high-occupancy water sites in order to analyse ligand binding modes, and iii) detect and describe tunnels and cavities. The analysis of water molecules’ distribution and trajectories sheds a light on proteins’ interactions with small molecules, on the dynamics of tunnels and cavities, on protein composition and also on the functionality, transportation network and location of functionally relevant residues. Finally, the correct placement of water molecules in protein crystal structures can significantly improve the reliability of molecular dynamics simulations.

## Graphical abstract

**Figure:**
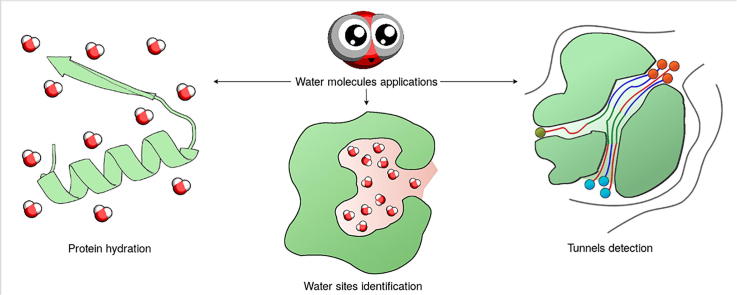

## 1 Introduction

Life began to evolve in an aqueous milieu, and the unique properties of water determine the chemistry of all living organisms. Water is a ubiquitous and essential substance in cells, accounting for about 70% of their mass. It is not only the environment for biological processes, but also an integral part of them [1]. At a macromolecular level, water contributes to biomolecules’ formation and their stability, dynamics and functions [2], [3], [4]. Water serves as a reaction reagent or mediates ligand–protein and protein–protein interactions. Water molecules are small enough to penetrate a macromolecule’s core, to stabilise its native structure and also to participate in processes occurring in the protein’s core [5], [6].

X-ray [7] and neutron diffraction [8] crystallography provide an insight into the spatial distribution of water molecules in the vicinity of biomolecular surfaces and confined regions such as active sites, pockets and cavities. Depending on the crystal quality, atomic resolutions can be achieved [9], [10], [11]. Protein structures deposited in the Protein Data Bank (PDB) [12] contain an abundance of information, i.e., alternative conformations of amino acid side chains and potential rearrangements of protein compartments. Information about water molecules’ positions is usually incomplete or can be strongly influenced by experimental conditions. Therefore, it is unclear how closely the distribution of crystal water molecules resembles the native conditions of the biomolecule. Nuclear Magnetic Resonance (NMR) is useful for discovering the hydration properties of proteins, especially their dynamics. Unfortunately, this technique cannot provide information about the three-dimensional structure of the hydration sites, and its time scales are shorter by an order of magnitude than the residence times of water molecules [4], [13].

The limitations of experimental methods can be overcome by computational techniques. *Ab initio* and DFT (Density Functional Theory) methods can be used for a precise description of a reaction mechanism, including the contribution of water [14], [15]. Molecular dynamics (MD) and Monte Carlo (MC) simulations provide a detailed atomic description of a biomolecule and a solvent, along with their dynamics [16], [17]. These simulations, however, do not tackle many equilibrium and long-time-scale kinetic properties [18].

The increasing awareness of the significant role of water molecules has given rise to a range of software focused on the analysis of water molecules’ behaviour. Recent reviews focused on virtual screening strategies describe several docking software applications that are capable of utilising information related to water molecules [19], [20], [21]. This paper provides a review of the available computational methods that employ water molecules for the analysis of macromolecules’ properties and structure dynamics. In the first part, we provide an overview of the techniques used for the prediction of water molecules’ locations. The following chapter describes the water sites that may participate in ligand binding. Next, water molecules are analysed in terms of ligand transportation and the detection of tunnels and cavities. For all three chapters, a list of software along with their functionality and/or their characteristics and principles is provided. The last chapter comprises conclusions and general remarks, as well as perspectives on the further development of software.

## 2 Software for protein hydration analysis

Water molecules not only maintain the functions of proteins but also stabilise their native structure [13]. The presence of water molecules in proteins’ internal cavities is conserved among homologous proteins families, as well as the key residues are [22]. It was shown, by reducing the amount of water during crystallisation [23] or by using mutants of particular proteins [24], that internal water molecules contribute to the structural folding and the stability of enzymes, ion channels, proton pumps and other macromolecules [25], [26], [27]. However, as we pointed out above, the experimental results are insufficient and can be inconsistent with each other [28]. The water molecules inside a protein’s structure may also be distorted, and their position may depend on the orientation of a particular side chain. They may also be trapped inside a protein’s cavity due to a process of large conformational changes.

The residence time of a water molecule buried in an internal cavity or trapped in a narrow cleft depends on its location and connectivity with the bulk solvent [29], [30]. Fast minimisation and short equilibrium stages can provide insufficient or inaccurate solvation of the protein interior and can bias the results. Therefore, it is important to fill the internal cavities with water molecules precisely, prior to running MD simulations. Lengthening the minimisation and equilibration procedure can provide sufficient exchange of water molecules between the surroundings and the protein interior; however, it is strongly system-dependent. Application of software developed to place water molecules into a protein’s cavities and its surroundings is proposed as an alternative strategy, especially for systems with large interior volumes, homology-modelled proteins or proteins with mutations introduced inside their cores (Table 1).

**Table 1:** List of available software to predict water molecules’ positions and orientation.

Software	Testing set	Accuracy*	Remarks
**Docking-based**			
*Dowser*[30]	14 crystal structures of OppA; D- and K-channels of cytochrome *c* oxidase; Photosystem II	63%	*Not available*
*Dowser+*[31]		74%	*Not available*
Dowser++ [39]		85%	Dowser++ standalone link
WaterDock [32]	14 crystal structures of OppA; HIV-1 protease; ribonuclease A; GluR2 ligand binding core; concanavalin A; glutathione S-transferase; carbonic anhydrase	88%	the code is available with the WaterDock 2.0 Pymol plugin: – link
WaterDock 2.0 [47]	14 crystal structures of OppA; HIV-1 protease; GluR2 ligand binding core; bovine pancreatic trypsin; glutathione S-transferase; HSP90; PIM1; series of 184 BRD4-BD1 complexes; androgen receptor; casein kinase II; thrombin; carbonic anhydrase	91%	WaterDock 2.0 standalone link WaterDock 2.0 Pymol plugin link

**RISM-based**			
*3D-RISM*[33]	*Alanine dipeptide; HIV-1 protease*	*–*	*Not available*
GAsol [34]	HIV-1 protease; neuraminidase; bovine pancreatic trypsin; series of 184 BRD4-BD1 complexes	94.3%	BSD 3-clause license link
Placevent [35]	HIV-1 protease; rotor ring of F-ATP synthase	water position error ~0.5 Å	the code and tutorial link

**Similarity-based**			
ProBis H2O [37]	*Src kinase* with bound bosutinib; human programmed death 1 (hPD) with ligand (hPD-L1); DNA Gyrase B; human matrix metalloproteinase (hMMP-1)	–	GUI – PyMOL integrated link
PyWATER [36]	thrombin; trypsin; BPTI; bromodomain-containing protein 4; MHC class I proteins; class A β-lactamases	identified all crystallographic water molecules	GUI – PyMOL integrated link

*Accuracy was calculated based on the number and quality of identified crystallographic water molecules. The numbers were taken from original publications. Please note that there are some differences in the details of the accuracy measurements. Information about currently unavailable software is in *italics*.

**Fig. 1:**
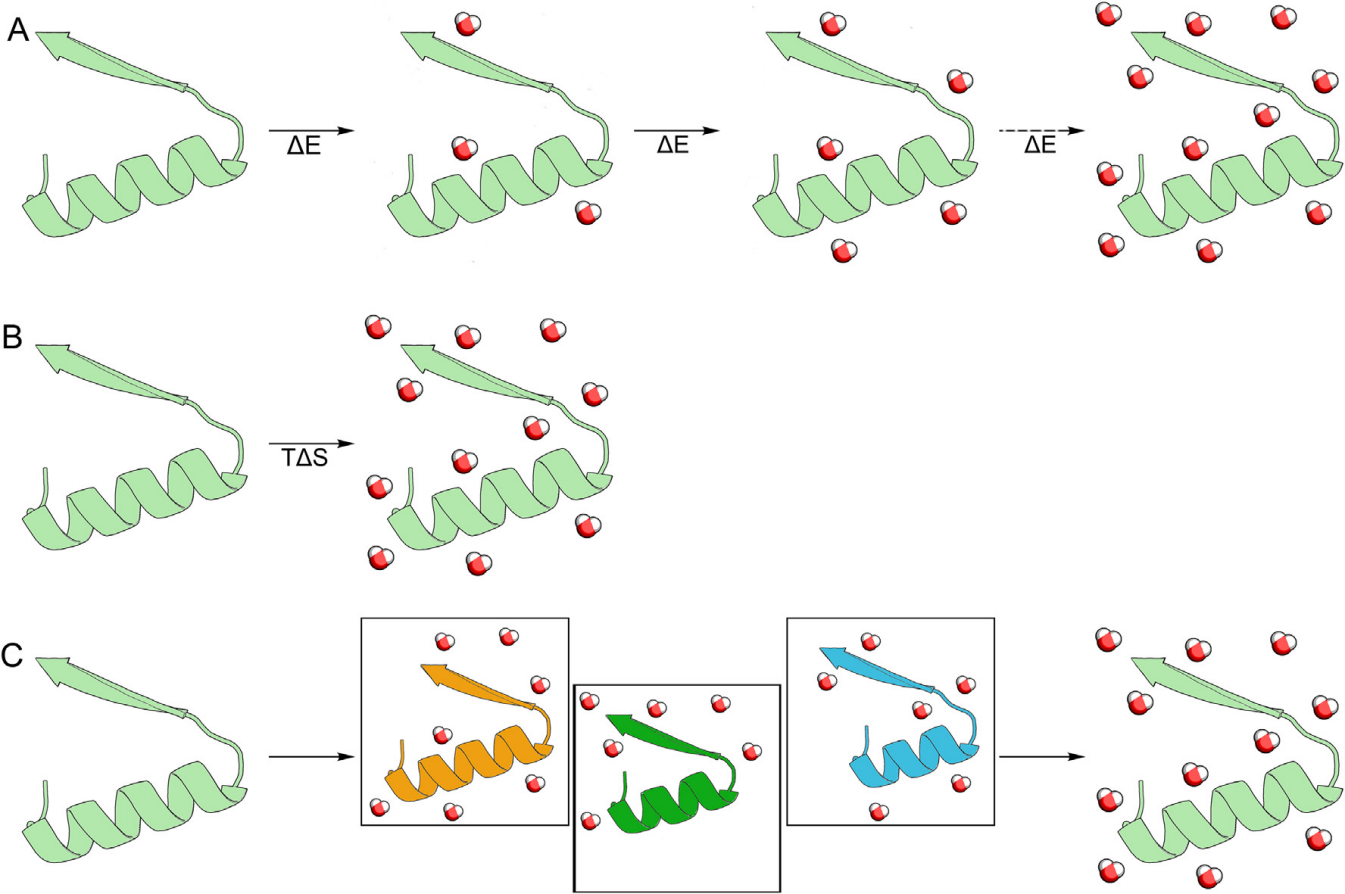
Strategies for placement of water molecules in the protein’s interior. (A) Docking-based, (B) RISM-based, and (C) similarity-based strategies.

Three different methods (Fig. 1) have been implemented for the placement of water molecules in a protein’s interior: i) based on the docking of water molecules, such as Dowser [31] and WaterDock [32], ii) based on the reference interaction site model (RISM), such as 3D-RISM [33], GAsol [34] and Placevent [35], and iii) based on the assumption that internal water molecules are conserved among similar proteins’ structures (PyWATER [36] and ProBiS H2O [37]).

The docking-based methods assume that the protein structure is the target and the water molecule is the ligand. Both Dowser and WaterDock utilise the commonly available docking software - AutoDock Vina [38]. These methods are fast and provide accurate positioning of the water molecules determined by crystallography. The water molecule docking algorithms have improved over new software versions, and for the Dowser software ‘generations’, the average accuracy of their predictions have increased from 63% in Dowser, to 74% in Dowser+ and up to 85% of the water molecules in Dowser++ [39], when compared to high-resolution crystallographic structures. WaterDock software presented a higher accuracy of crystallographic water molecules’ prediction than Dowser++: it was 88% for the original WaterDock and 91% for WaterDock 2.0; however, it should be kept in mind that there were some differences in the details of the accuracy measurements described in the original publications [39]. Along with their ability to predict water molecules’ positions, both WaterDock software releases are also able to determine if water molecules are displaced or ordered. WaterDock 2.0 comes with an easy-to-use PyMOL plugin.

The RISM theory is used for calculating the distribution of solvent molecules around a solute and has its roots in statistical, mechanical integral equation theories (IET) of liquids [34]. Due to the fact that the distribution calculated by 3D-RISM theory is continuous, it is difficult to directly examine specific solvent interactions, especially when they are numerous. 3D-RISM has been successfully used to locate water molecules in proteins as compared to experiment [40] and simulation [41], to calculate hydration free energies [42] and to predict fragment and drug positions [43]. The Placevent algorithm gave an average error for water molecules’ positions of about 0.5 Å [35]. The GAsol software, in which the 3D-RISM theory was combined with a genetic algorithm and a desirability function, showed the highest accuracy, with 93.4% of the predicted water molecules within 2 Å from their crystallographic positions [34]. Generally, RISM-based methods for water molecules’ prediction are slower than docking-based ones, and the computational time is system-size-dependent. However, they can be more accurate, especially for complex systems (e.g., metalloproteins, proteins equipped with large cavities or in complexes with nucleic acids) [44]. Moreover, it was shown that the RISM theory may break down in larger systems and systematically underestimates the partial molar volume (PMV) of amino acids [13].

As an alternative to the methods based on the physicochemical properties comes a simple similarity-based approach, implemented in PyWATER [36] and ProBiS H2O [37], which both superimpose crystallographic structures similar to the target protein and cluster the positions of conserved water molecules inside the protein cavities. ProBiS H2O is the first software that utilises the ProBiS algorithm [45] to perform local superimposition of the detected conserved water molecules. It also reduces the bias introduced by comparing similar protein structures or structures in different conformations than the query protein. PyWATER searches for similar structures using the PDB database [46]. The accuracy of such an approach strongly depends on the number, similarity and quality of related structures. Generally, ProBiS H2O gives fewer clusters with more tightly packed water molecules in comparison to PyWATER due to the clustering algorithms used (PyWATER uses hierarchical clustering, while ProBis H2O uses a Python implementation of 3D-DBSCAN (Three Dimensional Density-Based Spatial Clustering of Applications with Noise)). In addition, PyWATER stores information on the degree of conservation of each water molecule cluster with related atom numbers of water oxygen atoms from the superimposed protein structures.

All the tools mentioned above provide relatively fast, intuitive and accurate modelling of the water molecules in low-quality crystal structures and thus provide a more accurate starting point for an MD or MC study. Their usage can be also recommended for *in silico* prepared mutant structures, where substitution of residues enlarges or significantly reshapes internal cavities. However, the user should keep in mind that none of these methods assume flexibility or large conformational changes in the target structure. Also, in the case of preparing a very demanding protein structure, i.e., an unrefined homology model or a sole representative of a particular protein family, the user should be encouraged to avoid similarity-based or docking-based methods and focus on the RISM-based software to properly sample the positions of water molecules.

## 3 Software for water site detection and ligand binding analysis

Studies of protein–ligand interactions are crucial for a better understanding of the mechanisms of biological processes and their regulation [48]. Water-mediated interactions were found in 85% of 392 analysed protein–ligand complexes. Structural and thermodynamic data indicate that water molecules mediate interactions between proteins and ions, substrates, cofactors, inhibitors and other macromolecules [49], [50]. Water molecules are placed methodically within the surroundings and inside the protein, and display a particular structure characterised by the presence of hydration or water sites – regions of high-water density. They act as locations that attract water and can be used to describe water behaviour around chemical molecules [7], [51], [52]. The hydration/dehydration balance is relevant for protein–ligand formation and binding affinity, involving both entropic and enthalpic contributions [53], [54]. During the binding process, water molecules can either be displaced or conserved, bridging the protein–ligand interactions in the latter case [55]. The presence of water molecules in protein binding sites may imply different effects on the energy, entropy and enthalpy of the system. Depending on the situation, such effects may be favourable or unfavourable. For example, in a case where water molecules are trapped in a hydrophobic cavity filled by residues that cannot make appropriate hydrogen bonds, the enthalpic contribution is unfavourable. An opposite situation occurs when water molecules are engaged in forming hydrogen bonds to hydrophilic residues, and here the enthalpic effect may be favourable [56]. The displacement of such water molecules can contribute to the binding free energy, impact affinity during ligands’ association and govern enthalpy and entropy partitioning, according to the properties of the individual water molecules compared to those in the bulk phase [49]. Developing a ligand with a high binding affinity towards a specific target is one of the most important steps during the entire drug design process. Thus, a lot of effort is focused on the prediction of whether a water site should be displaced and whether this would cause an increase in a ligand’s affinity.

**Fig. 2:**
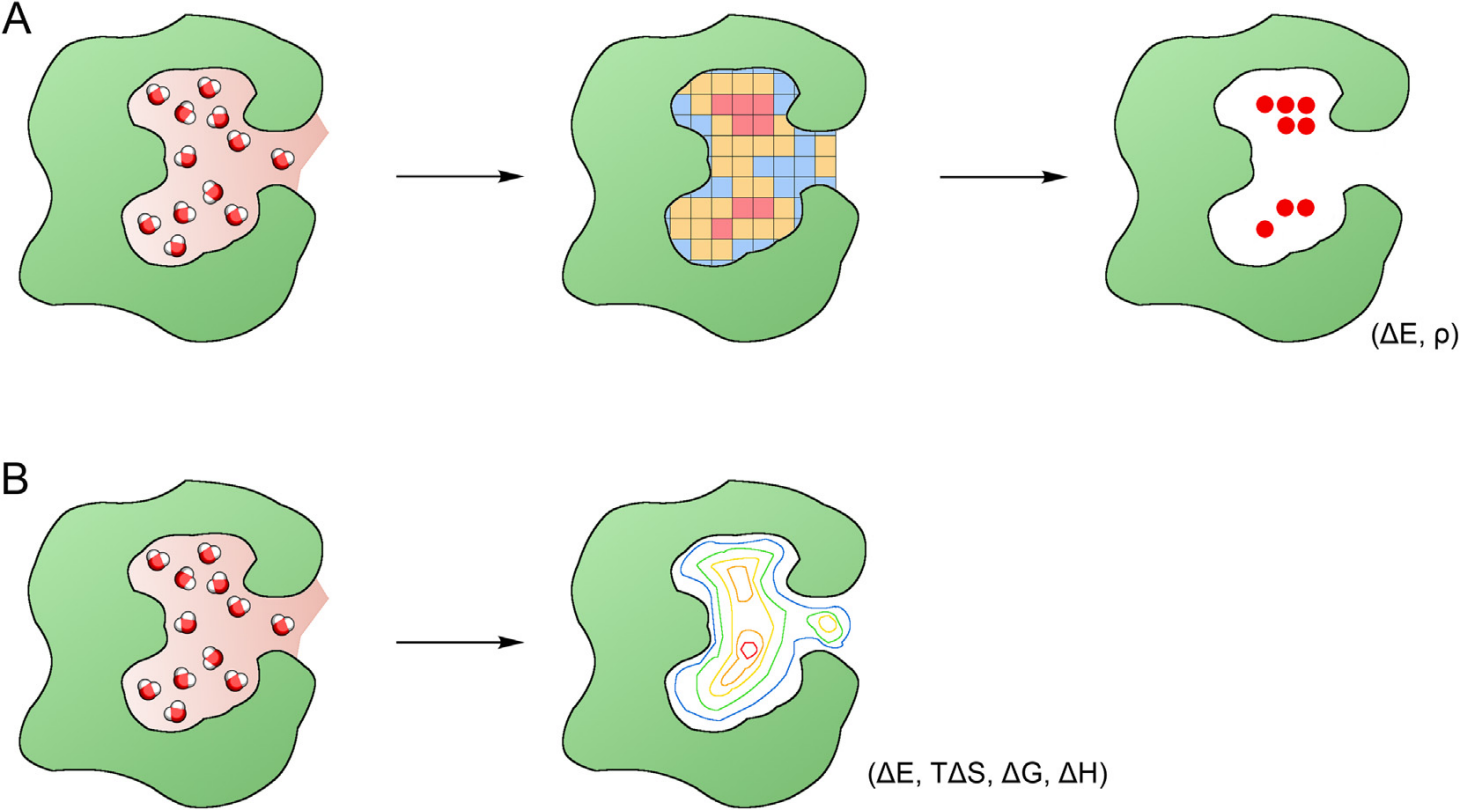
Strategies of analysis of water sites and ligand binding modes. (A) Strategy using a grid to calculate energetics based on water local distribution, and (B) strategy using IFST (Inhomogeneous Fluid Solvation Theory) to assess the role of structural water molecules by calculating their contribution to the thermodynamics of protein solvation. Grid cells (squares at row A) are coloured according to increasing number of water molecules detected in cells (green – low, red – high). Cells with highest occupancy provide information about the energetically preferred position of the water molecules. Calculated isolines (row B) provide information about the same values of the thermodynamic factors calculated for water molecules in protein cavities. (For interpretation of the references to colour in this figure legend, the reader is referred to the web version of this article.)

Different approaches, both experimentally-based (i.e., location in crystal structures and B-factors) and knowledge-based (i.e., free energy, water’s contribution to binding free energy, entropic contribution), have been reported to assess information about water sites [55]. One of the very first experimentally-based software programs, GRID [57], uses a regular array of ‘grid points’, established throughout and around the protein, to calculate the energetics of water probes inside a macromolecular binding site (Fig. 2a). GRID places a chemical probe and calculates an empirical interaction energy at all grid points [55]. An approach using crystallographic B-factors to determine which water molecules in a protein’s structure are likely to be displaced has been implemented in Consolv [58] and WaterScore [59] software. Using geometric criteria can also indicate the positions of water molecules mediating protein–ligand interactions. Such a procedure has been included in the AcquaAlta program [60] for estimating the propensities of ligand hydration. In HINT software [61], the Gibbs free energy of non-covalent interactions is based on van der Waals interactions and partial atomic partition coefficients. A knowledge-based approach has been implemented, e.g., in AQUARIUS [62] or AQUARIUS2 [63] software. The probable positions for hydration sites are predicted based on solvent distributions surrounding particular amino acids derived from the analysis of a protein’s structure. However, most of the software applications mentioned above are not currently used or are used very rarely. This is due to the fact that another class of methods, describing the thermodynamic properties of water by analysing data from MD and MC simulations, became very popular and easy to use.

Most of the recently developed tools are based on Inhomogeneous Fluid Solvation Theory (IFST) derived in 1998 by Lazardis [64]. IFST is a statistical mechanical method that calculates free energy differences from short MD or MC simulations by quantifying the effect of a solute acting as a perturbation to bulk water. The solute may be different molecules, such as proteins, peptides or other molecules. One of the major advantages of IFST is that the system is spatially decomposed to consider the contribution of specific regions to the total solvation free energies. The contributions of each individual water molecule to the enthalpy are calculated by computing the average interaction energies, whereas the contributions to the entropy are calculated from intermolecular correlations. The Gibbs free energy equation can be used to calculate the contribution to the free energy from the enthalpy and entropy. The result is then compared with the contribution of one water molecule to the free energy of bulk water, again calculated using IFST. The results of IFST depend on the accuracy of the force field and the water model that was used: a detailed discussion can be found elsewhere [65], [66]. IFST is implemented in WaterMap [67], GIST [68], WATCLUST [69], STOW [70] and WATsite [71] software. Methods based on IFST are limited to the analysis of high-occupancy hydration sites and therefore omit solvent molecules found in lower density regions [68] (Fig. 2b).

Some of the software listed in Table 2 perform calculations based on static input (WATsite, WATGEN [72], FLAP [73], WaterFLAP [74], SZMAP [75], WRAPPA [76], WaterScore [59], AcquaAlta [60], JAWS [77] and WScore [78]), while some of them rely on data obtained from MC or MD simulations (AQUA-DUCT [79], [80], GIST [68], WATCLUST [69], STOW [70], SSTMap [81], WaterMap [67], Water-swap [82], WatMD [83], SPAM [84] and AquaMMapS [85]). In static structure-based software, hydration and water sites are determined by investigating potential binding sites through the placement of water probes. The only exception is WATsite, which conducts MD simulations based on the static input structure. In simulation-based software, the system is simulated with explicit water molecules, which are free to explore the system’s space. These water molecules are then clustered in hydration sites, and their thermodynamic properties are calculated. Some of the software only identifies the water sites, without any further information, while some can estimate the binding free energy and the corresponding thermodynamic components for water molecules in the binding sites. In a very recent paper, the authors combined WATsite data with neural networks and deep learning to significantly improve the speed of water site identification and the calculation of the free energy contributions [86]. The authors claim that such an approach will allow the inclusion of solvation components, such as water-mediated interactions or enthalpically stable hydration networks in proximity to the protein–ligand complex, in structure-based ligand design.

**Table 2:** List of software for analysis of ligand binding and drug design with respect to the water molecules in the binding cavity.

Software	Testing set	Functionality	Remarks
**Input – a single structure**			
AcquaAlta [60]	trypsin; dihydrofolate reductase; thymidine kinase; VEGFR2; glycogen phosphorylase; human phosphodiesterase; beta trypsin; holo-glyceraldehyde 3P dehydrogenase; HSP90; AmpC beta-lactamase; 2CDK2; ACE; COMT; HIV-1 protease; non-nucleoside adenosine deaminase; ACK1; coagulation factor Xa; EGFR	generating of explicit water molecules at the ligand–protein interface; searching for water molecules interacting with generic functional groups of small organic molecules; generating water molecules bridging interactions between ligand and protein considering the hydration propensities of the involved functional groups and aromatic moieties	available on request: link
FLAP [73]	a set of 23 protein kinase structures	target-based pharmacophores; comparison of multiple protein targets; docking ligands into protein targets	commercial, standalone: link
JAWS [77]	neuraminidase; scytalone dehydratase; Major Urinary Protein 1; bovine β-lactoglobulin; cyclooxygenase-2	determining the optimal placement of water molecules in a binding site; binding free energy estimation	implemented in a modified version of MCPRO, v. 2.1 [87]
SZMAP [75]	HIV-1 protease; neuraminidase; trypsin; factor Xa; scytalone dehydratase; oligopeptide-binding protein (OppA);	computation of binding free energies and the corresponding thermodynamic components for water molecules in the binding site	commercial link
WaterFLAP [74]	adenosine A_2A_ StaR receptor in complex with triazine	generating and scoring water networks for both apo and ligand-complex structures; binding path prediction; lipophilic hot-spot calculation	commercial, standalone: link
*WaterScore*[59]	*cutinase; xylose isomerase;* *penicillopepsin; galactose/glucose binding protein; proteinase A; rhizopus pepsin; actinidin; DNase I; cholesterol oxidase; RNAse A; thermitase; lipid binding protein; Fv fragment of mouse monoclonal antibody D1.3; dihydrofolate reductase*	*determine conserved water molecule positions; scoring of protein–ligand interactions and determination of ligand binding mode with respect to bound and displaced water molecules*	*link**(currently unavailable)*
*WATGEN*[72]	*126 protein–peptide binding interface structures*	*identification of water sites; selection of the ‘best’ water sites for ligand docking; solvation thermodynamics; binding free energy estimation*	*no information available*
*WATsite*[71]	*three different structures of protein–ligand complexes of factor Xa*	*identification of water sites; free energy estimation*	*link**(currently unavailable)*
WRAPPA [76]	vinculin binding-site; truncated SNARE complex; potassium channel fragment; human relaxin-3; RNA complexed with Rev peptide; Kv1.3 channel blocker Tc32	identification of water sites, referred to as dehydrons	web server: link
*WScore*[78]	*a set of 542 binding sites within 506 protein–ligand complexes, associated with 22 receptors*	*predicting the location of water sites; producing a detailed map of the water structure and displacement free energies; ligand docking and scoring*	*no information available*

**Input – MDsimulations**			
AQUA-DUCT [79], [80]	*Solanum tuberosum epoxide hydrolase*[88]	*high-density water sites’ and/or co-solvent sites’ identification*	*standalone:**link*
*AquaMMapS*[85]	casein kinase 2; A_2A_ adenosine receptor	identification of spatial hot spots within the protein binding site	no information available
GIST [68]	Cucurbit[7]uril (CB7); factor Xa	high-density water sites’ identification; map of regions where the solvent interacts favourably with the surface or has unfavourable entropy	implemented in AmberTools
SPAM [84]	HIV-1 protease; hen egg-white lysozyme	qualitative estimation of the thermodynamic profile of water in hydration sites; binding free energy estimation	implemented in AmberTools
SSTMap [81]	Caspase 3	identification of water sites	link
*STOW*[70]	*HIV-1 protease-ligand complex;* *concanavalin A-carbohydrate complexes;* *cyclophilin A-ligand complexes*	*computation of contribution of discrete ordered water molecules to the solvation thermodynamics; determine and analyse water sites*	*no information available*
WATCLUST [69]	AmpC beta-lactamase	determine and analyse water sites	VMD plugin: link the direct transfer of the information to Autodock
Water-swap [82]	neuraminidase in complex with oseltamivir	calculation of binding free energy by water-swap reaction coordinate	part of the Siremol’s Sire application: link
WaterMap [67], [89]	streptavidin; Cox-2; antibody DB3; HIV-1 protease	identification of water sites; solvation thermodynamics; entropic and enthalpic contributions to the free energy	commercial, part of the Schrödinger package: link
*WatMD*[83]	*Green Fluorescent Protein;* *Mannitol 2-Dehydrogenase*	*identification of water sites*	*no information available*

*Information about currently unavailable software is in *italics*.

## 4 Software for tunnel detection and transportation phenomena analysis

The intramolecular voids inside a protein structure, such as cavities, tunnels, channels and pores, are often important for protein functions [90]. While we have already shown the importance of cavities, this section focuses on the function of tunnels and channels. For proteins with a buried active site, tunnels facilitate substrate entry and enable product egress. Tunnels, as well as the whole protein structure, should not be seen as rigid bodies. In fact, a reasonable degree of flexibility is often required to maintain the catalytic reaction. The geometry and amino acid composition of a particular tunnel determine the shape and chemical properties of a potential ligand. Tunnels are also equipped with a much more sophisticated mechanism of small molecule discrimination – gates. Gates are capable of controlling substrate access to the active site, preventing solvent access to particular protein regions and synchronising processes occurring in distant parts of the protein [91]. Tunnels, pores, gates and cavities constitute a dynamic network inside a protein. Therefore, for proper tunnel detection, a single crystal structure of a protein may be insufficient. MD simulations provide a picture of a protein’s movements in an aqueous solution. Reasonably long simulations give insights into the dynamics of the tunnel network. Well-defined tunnels allow fast water exchange over a time of about 10^−9^ s, while transient tunnels extend the required time up to 10^−3^ s. In comparison, the exchange time of water molecules at the protein surface with bulk ones is in the sub-nanosecond range [92]. Therefore, from the computational point of view, the lengths of the required molecular dynamics simulations depend on the studied system. In the case of buried active sites linked with the solvent *via* a network of tunnels, hundreds of nanoseconds are enough to provide good sampling [88], [93], [94], [95], [96], whereas to observe the exchange of deeply buried water molecules with the bulk solvent up to as much as tens of milliseconds are necessary [30]. Shorter simulations can provide information about the potential pathways of such an exchange and can suggest mutations that can open an alternative tunnel [80]. The second parameter which might influence the required length of simulations is the frequency of gating phenomena. Gates defined by a single amino acid’s rotation require shorter experiments than those defined by, e.g., loops or controlled by proteins’ breathing motions [91].

The first tunnel detection software used a geometry-based approach to identify ‘empty spaces’ inside protein structures [90]. The most successful ones, such as Mole 2.0 [97], CAVER 3.0 [98], and CAVER Analyst 1.0 and 2.0 [99], [100], are widely used by the scientific community, predominantly to describe tunnels identified in crystal structures. The most successful strategy employs the construction of a Voronoi diagram to detect and describe voids within the macromolecule [101], [102]. Using a defined probe radius and internal cavity identification, the software is able to detect tunnels providing access from the selected area to the surroundings. Such a strategy assumes that the tunnels are a summation of connected cavities and is very often used for the analysis of single crystallographic structures. The structural information obtained on such a basis is mostly incomplete, due to tunnels’ flexibility. Moreover, using spherical probes for tunnel exploration provides only an approximation of tunnels to tubes with symmetrical diameters and thus prevents analysis of tunnels’ asymmetry. It is also difficult to analyse the regulation and direction of the solvent flow, as well as the contribution of tunnels to an enzyme’s activity and selectivity. Some of these weaknesses were targeted in 2014 by a non-spherical approach by Benkaidali et al. [103]; however, due to its complex implementation the tool was rarely used. Results provided by geometry-based tunnel detection software were unable to answer questions about solvent flow direction and how tunnels contribute to this.

**Fig. 3:**
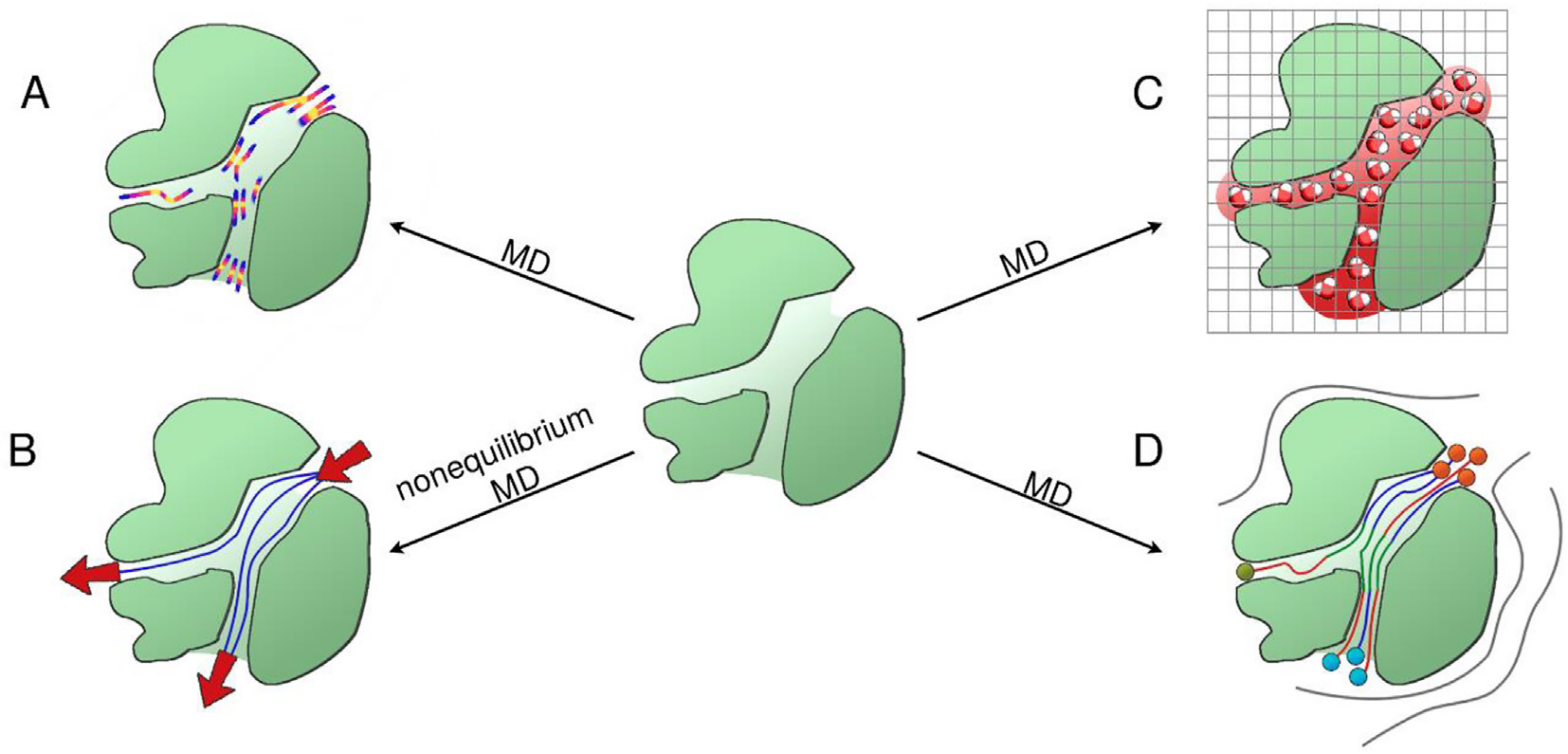
Strategies for tunnel detection and description based on water molecule analysis. (A) Streamline tracing, (B) Solvent Flux, (C) *trj_cavity*, and (D) AQUA-DUCT water tracking approach.

To analyse the solvent flow direction and tunnels’ contribution to this parameter, we need to concentrate on solvent/ligand analysis. Several different methods have been implemented, based on very diverse approaches (Fig. 3). The first attempt was made in 2008 by Bidmon et al. [104], who introduced the Visual Abstractions of Solvent Pathlines method. The pathways of solvent molecules passing through the particular region of interest (so-called ROI) were pre-processed and visualised as Bézier curves. The next attempt at such an analysis was carried out in 2010 by Vasiliev et al. [105]. Their streamline tracing method was applied to photosystem II and was used to visualise water flux in particular regions of the protein. However, the results of the calculations are hard to interpret, and only a few applications of this method can be found in the literature [106]. In 2014, Benson and Pleiss proposed a solvent flux method to study water influx in the *Candida antarctica* lipase B protein cavity from the triglyceride-water interface [107]. They introduced a solvent concentration gradient and the reorientation and rescaling of the velocity vectors of selected water molecules in order to accelerate the influx and increase the probability of rare events in the study. Similarly to widely used strategies (aMD, REMD, SMD and RAMD), it was applied to investigate rare events in a reasonable computing time range (e.g., it overcame the significant energy barriers of slow biophysical events). In contrast to known methods, this technique allowed the flow of multiple molecules, including the selected solvent molecules, to be precisely investigated during a single simulation. Since artificial external forces are introduced to classic MD simulations, one could be concerned about misleading biases and the complicated protocol that is dependent on the system. In 2014 another software program emerged as a GROMACS plugin, called *trj_cavity* [108]. *Trj_cavity* is capable of cavity and tunnel identification together with time-dependent calculations of their volume and solvent capacity. In the vast majority of research papers, *trj_cavity* is used only for the identification of cavities and calculating their volumes and occupancy, while only one study was found where the authors used *trj_cavity* to actually trace ligands [109]. The existing gap between tools searching for tunnels and pathways, and advanced tools for accelerated water flux investigations was filled in 2017 by AQUA-DUCT [79], an easy-to-use tool facilitating analysis of the behaviour of water (and, if necessary, other solvent molecules) penetrating any selected region in a protein. AQUA-DUCT comprises a *Valve* module, which is capable of tracking water molecules, clustering their trajectories and enabling visualisation in PyMOL [110]. The *Valve* module was used to investigate relatively small proteins, such as D-amino-acid oxidase (DAAO) [111] and *Pyrococcus furiosus* phosphoglucose isomerase [93], as well as ion channels such as claudins [112]. In contrast to the geometry-based approach, water tracking analysis provides information about tunnels’ functionality, allows their permeability to be compared and facilitates the detection of the gating residues controlling access to the binding cavity. At the same time, Watergate, a software application for statistical overview of the overall solvent flow, water trajectory clustering, and visualisation was developed [113]. The software programs using water molecules for tunnel detection are listed in Table 3.

**Table 3:** List of software and methods for tunnel detection and transportation phenomena observation.

Software or method	Applicability		Remarks
	System	Functionality	
AQUA-DUCT [79], [80]	*Mus musculus* epoxide hydrolase	water molecule tracking	can be used also for cavity and hot-spot detection Standalone: link
	d-amino-acid oxidase [111]	tunnel and gating residue detection	
	*Pyrococcus furiosus* phosphoglucose isomerase [93]	water molecule tracking and occupancy analysis in the internal cavity	
	Claudin-2 ion channel [112]	ion transportation pathways identification	
*Solvent flux method*[107]	*Candida antarctica lipase B*	*identification of water access pathway; hot-spot identification*	*based on an artificial gradient. Code not available.*
Streamline tracing [105]	photosystem II	fibre tracing; tunnel detection; gating residue (access control points) identification	visual analysis only; code available on request
	squalene–hopene cyclase [106]	changes in water flow after introducing amino acid substitution	
**trj_cavity**[108]*	Der p 2 protein; TM pore; pullulanase	generating the trajectory of discovered cavities, quantification of time-dependent cavity volume, solvent presence inside a particular cavity; tunnel detection	implemented in GROMACS: link
	polydicyclopentadiene [114]; herkinorin [115]; glycidoxypropyltrimethoxy silane [116]; mammalian translocator membrane protein [117]; human G-protein coupled receptors [118]; amyloid fibrils [119]; sperm whale myoglobin [120]; 07A metalloprotease [121]; human erythrocyte anion exchanger 1 (Band 3 protein) [122]; amorphous silica [123]; full-length TLR4 dimer [124]; profilin [125]; human serum albumin [126]; laccase [127]; dengue capsid protein (C protein) [128]; horseradish peroxidase; lactoperoxidase [129]; OmpC–MlaA complex [130], [131], [132]; MATE transporter [133]	cavity analysis	
	cholesteryl ester transfer protein [134]; acyl carrier proteins [135]	time-dependent cavity analysis	
	mouse myoglobin [109]	analysis of the movement of ligands, movements within the cavities and tunnels of proteins	
*Visual Abstractions of Solvent Pathlines*[104]	*TEM β‐lactamase*[136]	*identification of the role of water in gating loop flexibility*	*visual analysis only; code not available*
Watergate [113]	haloalkane dehalogenase mutants	visualisation of water molecule trajectories	visual analysis only; code available on request

*For *trj_cavity* software only recent applications are presented (2017–2019). Information about currently unavailable software is in *italics*.

Tools based on water molecules as a molecular probe for tunnel detection (listed in Table 3) can provide much more complex information about proteins than simple geometry-based methods. Since they are focused on the information provided by the solvent itself, they also take into account the physicochemical properties of the solute. Such information is useful for examining the effects of the introduced mutation on the solvent flow and thereby the enzyme’s activity. By using the pathways of the solvent molecules, the user is able to identify the key residues important for the enzyme’s activity and selectivity, and the amino acids that contribute to gating residues and control small ligands’ entry/egress. These tools can additionally facilitate the description of cavity shape evolution during simulation time, which can be used for inhibitor design or hot-spot detection for substrate specificity modification, and also the identification of residues distant from the active site which contribute to the activity and selectivity, and thus can be considered as a safe alternative to smart mutant library design. However, it should be kept in mind that to properly sample events such as substrate entrance, product release or the exchange of the trapped solvent molecules with the bulk solvent, the analysed simulations must be of reasonable length and conducted in physiological-like conditions (please see the Summary and outlook section for more details).

## 5 Summary and outlook

The important role of water molecules in structural biology is reflected by a large number of different software programs dedicated to various types of water-molecule-based analysis. Most of the software presented here is focused only on particular aspects of water’s presence in a macromolecular structure, such as its contribution to protein stability, ligand binding and drug design, or cavity and tunnel description.

Among all the described software, the role of software in optimising water placement inside proteins’ cavities is probably the most underestimated, although placing water molecules is not a trivial task. Three different strategies, RISM theory, the docking of water molecules, and the analysis of conserved water molecules among similar proteins, are used and complement each other. The RISM-based software probably provides the most accurate model; however, it is time-consuming. Docking-based methods are the fastest; however, they may provide biased results for systems, such as metalloproteins, proteins with large cavities and protein-nucleic acids complexes, that are problematic for such software. Both methods can be considered for the prediction of differences in water rearrangement when mutant structures are designed. The third strategy requires a collection of similar structures and may not be sensitive enough to provide correct predictions when a single mutation occurs. Therefore, depending on the investigated system, different strategies are optimal for water placement and can provide reliable starting points for molecular dynamics studies. As already stated, the water placement method should be considered as a standard approach for homology models or structures with introduced amino acid modifications.

Concerning the role of water in the description of ligand binding, the most commonly used methods are based on IFST. Given a water site, the software can predict how much free energy is gained (or lost) by displacing the water molecules that occupied the potential ligand-binding site. The solvent contribution cannot be neglected, as was shown in several excellent papers [137], [138], [139], and therefore continuous progress in both the accuracy and parallel analysis of alternative states is highly desired. Binding enthalpies and entropies of water molecules may also be calculated based on Grid Cell Theory (GCT) [140]. This is a recently developed method for investigating hydration thermodynamics from a molecular dynamics or Monte Carlo standpoint. In the GCT approach, the density, enthalpy, entropy and free energy of water are evaluated for an arbitrary region of space around a system of interest. These parameters refer to the water molecules which enter a particular hydration site of the protein(s) from the bulk concentration. However, this theory has not yet been implemented in known software. The recent AQUA-DUCT version provides an approach combining information on water and co-solvent high-occupancy sites. It can be used for pharmacophore design and suggests directions for future software development. All of the abovementioned methods can provide additional support to drug design and provide more accurate results in comparison to methods neglecting water molecules’ contribution.

In the third group of software, water molecules are used as a molecular probe to sample the ‘empty spaces’ in proteins during molecular dynamics simulations to detect tunnels and cavities. This field is so far monopolised by a widely used geometry-based approach which on the one hand is very simple, but provides rather approximate results. It neglects the tunnels’ asymmetry and the physicochemical properties of the tunnel-lining amino acids. Therefore, it is difficult to use such software for analysis of tunnels’ functionality. The alternative approaches presented in our review are a most diverse group of software utilising water as a molecular probe. Depending on the implemented algorithms, they provide information about local water flow changes (such as the streamline tracing method), changes in cavity volumes (*trj_cavity*) or can provide a holistic picture of water flow *via* tunnel networks and an approximation of the energy profiles of particular pathways (AQUA-DUCT). The utilisation of water for cavity and tunnel description removes most of the limitations of the standard approach. It seems that using water molecules as a molecular probe enables more sophisticated analysis of the substrate transportation network provided by tunnels, handling tunnels and cavities together and describing the protein interior in a holistic way as a single entity. However, so far it is hard to provide an estimation of how accurate they are. This problem is caused not only by difficulties in experimental verification of their findings, nor the question of how accurately the hydrophobic cavities can be described, but also due to the lack of benchmarks for the performance inside protein structures of the different water models used in MD simulations.

Protein engineering is one of the most promising, but still largely unexplored, fields of application for software focused on the analysis of water as a molecular probe. So far, most of the examples of such studies are focused on understanding the changes introduced by mutant proteins’ construction. However, there are papers showing the potential applicability of the water-based approach for hot-spot detection [88] and mutant library design [111]. Successful verification of such a strategy can greatly facilitate protein engineering and provide an interesting and easy-to-apply technique. Also, using the information on the water molecules’ (or small ligands, or other types of solvent molecules) tracking, the user gains knowledge on a protein’s internal architecture, which might be used to develop a successful strategy for further modifications; for instance, to search for more potent inhibitors which will explore previously unused cavities, or to improve the protein’s activity and/or selectivity by adjusting the pathways leading to and from the active site.

Since the analysis of water-mediated interactions has become of greater interest, we hope that the number and quality of software programs using water molecules to analyse macromolecules’ properties will only increase. However, progress in this promising area cannot be achieved without the further joint efforts of theoreticians and experimentalists. One needs to consider that the majority of the tools described above depend on water models and force fields (e.g., tools based on Inhomogeneous Fluid Solvation Theory or benefiting from MD simulations). Both force fields and water models are being constantly upgraded to provide more accurate descriptions of studied systems. For example, the most recent papers of Huang, et al. provide force fields which can be used for both ordered and disordered proteins [141]. Recent four-point water models have improved the description of its thermodynamic properties; however, water molecules’ non-bonded interactions still require validation [142]. Nevertheless, the majority of computational studies employ simple non-polarizable models of water (e.g., TIP3P, SPC/E, TIP4P) and assume that they will describe water molecules in macromolecular surroundings equally well as in the bulk water. Unfortunately, there is no study that can confirm such a presupposition, simply due to the limited access to experimental data providing insight into water’s behaviour inside a protein’s core. Moreover, even the benchmark analysis of a particular software data’s dependency on the used parameters is very limited. As we mentioned above, the comparison of the IFST results obtained with different water models suggests that the quantitative application of IFST to biological systems is strictly model-dependent and has to be carefully analysed. Fortunately, several successful verifications of the findings guided by the software developed to analyse the behaviour and/or properties of water molecules have accelerated research in the field of protein research and each year bring to the scientific community new, optimised, versatile and reliable tools which greatly improve our understanding of nature.

## Funding

This work was funded by the National Science Centre, Poland, grant no DEC-2013/10/E/NZ1/00649 and DEC-2015/18/M/NZ1/00427.

## Conflict of interest

None declared.

## CRediT authorship contribution statement

**Karolina Mitusińska:** Resources, Data curation, Writing - original draft, Writing - review & editing, Visualization. **Agata Raczyńska:** Resources, Data curation, Writing - original draft, Investigation, Visualization. **Maria Bzówka:** Resources, Data curation, Writing - original draft, Investigation, Writing - review & editing. **Weronika Bagrowska:** Resources, Data curation, Writing - original draft, Investigation. **Artur Góra:** Conceptualization, Supervision, Funding acquisition, Project administration, Writing - review & editing.
